# Selection of Lactic Acid Bacteria with In Vitro Probiotic-Related Characteristics from the Cactus *Pilosocereus gounellei* (A. Weber ex. K. Schum.) Bly. ex Rowl

**DOI:** 10.3390/foods10122960

**Published:** 2021-12-01

**Authors:** Karoliny Brito Sampaio, Thatyane Mariano Rodrigues de Albuquerque, Noádia Priscila Araújo Rodrigues, Maria Elieidy Gomes de Oliveira, Evandro Leite de Souza

**Affiliations:** 1 Laboratory of Food Microbiology, Department of Nutrition, Health Sciences Center, Federal University of Paraíba, João Pessoa 58051-900, Paraíba, Brazil; karolbsampaio@gmail.com (K.B.S.); thaty_mra@hotamil.com (T.M.R.d.A.); noadia_priscila@hotmail.com (N.P.A.R.); 2 Laboratory of Bromatology, Department of Nutrition, Health Sciences Center, Federal University of Paraíba, João Pessoa 58051-900, Paraíba, Brazil; mego@academico.ufpb.br

**Keywords:** xique-xique, unconventional plant food, probiotics, technological properties, survival, physiological functionalities

## Abstract

*Pilosocereus gounellei* (A. Weber ex. K. Schum.) Bly. ex Rowl., popularly known as xique-xique, is a cactus from the Caatinga biome, which is rich in bioactive compounds but has not been previously studied as a source of lactic acid bacteria (LAB) with probiotic aptitudes. This study aimed to identify, characterize, and select LAB isolates with in vitro probiotic-related characteristics from xique-xique cladodes and fruit. Isolates with the most promising probiotic-related characteristics were evaluated regarding their in vitro technological properties and capability of surviving in chestnut milk, whey protein drink, and mate tea with mint during 21 days of refrigeration storage. Seventeen recovered isolates had typical characteristics of LAB. Six out of these seventeen LAB isolates passed the safety tests and were included in experiments to evaluate the in vitro probiotic-related characteristics. Based on the results of a principal component analysis, the isolates 69, 82, 98, and 108 had the best performances in experiments to evaluate the probiotic-related characteristics. In addition to showing good technological properties, the four selected LAB isolates had high viable counts (>7.3 log cfu/mL) and high sizes of physiologically active cell subpopulations in chestnut milk, whey protein drink, and mate tea during refrigeration storage. These four isolates were identified by 16S-rRNA sequencing as being *Lacticaseibacillus paracasei* or *Lacticaseibacillus casei*. The results indicate xique-xique as a source of potentially probiotic LAB isolates.

## 1. Introduction

In recent years, the consumption of probiotic foods gained increasing attention as an alternative for health promotion [1]. Probiotics are live microorganisms capable of conferring a health benefit to the host when administered in adequate amounts [2,3]. The efficacy of the supplementation of probiotic lactic acid bacteria (LAB) to prevent and/or treat various pathologies, such as obesity, irritable bowel syndrome, inflammatory bowel, and allergic disease, has been widely reported and recognized as a strain-dependent feature [2,3,4]. 

The discovery of new candidates for use as probiotics led to the search for strains from the local biodiversity [3,4]. The selection of LAB with probiotic-related aptitudes for use in foods must include the evaluation of safety, physiological functionality, and technological aspects, as well as their survival in food matrices intended for delivery [3]. Plants, vegetables, and fruit harbor enormous genetic potential of valuable but still undiscovered LAB strains. Although LAB is only a small part forming the plant autochthonous microbiota, these microorganisms represent the most important microbial group with the capability of promoting beneficial alterations in the health-promoting properties of plant foods [5,6]. 

Early studies showed a high diversity of potentially probiotic endophytic LAB from cladodes and cactus fruit. However, these investigations were largely related to the genus *Opuntia* [6,7]. *Pilosocereus gounellei* (A. Weber ex. K. Schum.) Bly. ex Rowl., popularly known as xique-xique, is most used by the population of the Brazilian semiarid region [8]. It is a non-conventional plant food from the Caatinga biome with rich nutritional composition and presence of several health-related bioactive compounds [9]. Xique-xique cladode juice exerts selective stimulatory effects on the growth and metabolism of different probiotic *Lactobacillus* species known as part of the human intestinal microbiota to the detriment of enteric bacteria, indicating prebiotic properties [10]. Additionally, xique–xique fruit is a rich source of phenolic compounds and betalains, besides having a strong antioxidant capacity [11]. However, no previous study evaluated the potential probiotic properties of autochthonous LAB from xique-xique. 

Over the years, the global market for probiotic non-dairy alternatives (or milk analogues) progressively increased [12], especially the development of probiotic products using plant-based matrices nutritionally balanced and/or with added value [13]. Since the efficacy of probiotics is partially associated with their capability of surviving in the carrier food products, several approaches have been considered to maintain the viability of probiotic cells in these vehicles, including the proper selection of probiotic isolates and suitable food matrices for their delivery [14]. LAB isolates from vegetable origin have stood out in regard to their probiotic properties, probably because they are commonly more resistant to the gastric environment than their counterparts of animal origin, indicating a promising potential for the use of vegetables as a source of probiotic microorganisms [15].

This study has hypothesized that xique-xique could be a source of LAB isolates with the potential to be exploited as novel probiotics. To test this hypothesis, this study identified, characterized, and selected LAB isolates from xique-xique cladodes and fruit with characteristics compatible for use as potential probiotics. The LAB isolates selected as having the most promising in vitro probiotic-related characteristics were evaluated regarding some in vitro technological properties and their capability of surviving in different food matrices during refrigeration storage with measurements of viable counts and cell physiological states.

## 2. Materials and Methods

### 2.1. Isolation and Preliminary Identification of LAB

Xique-xique (*Pilosocereus gounellei*) cladodes and fruit (green and ripe) were collected in a private growing area (Boa Vista, PB, Brazil; 07°15′32″ S 36°14′24″ W). The samples were selected considering their physical integrity and transported in polystyrene boxes under cold temperature (10 ± 1 °C). The material was botanically identified by Prof. Dr. Leonardo Person Felix (Center of Agrarian Sciences, Federal University of Paraíba, Areia, PB, Brazil). The certified species was deposited in the Prof. Jaime Coelho Morais Herbarium (Center of Agrarian Sciences, Federal University of Paraíba) under access number 17.562. The plant material collection was recorded in the Information and Biodiversity System of Brazil (SISBIO; number 62681), as well as in the National System for Management of Genetic Heritage and Associated Traditional Knowledge (SISGEN; number AA17429).

The preparation of cladode and fruit samples involved washing them in potable running water, disinfecting them by immersion in chlorinated water (100 ppm) for 15 min, and rinsing them with potable water. The cladodes were divided into two structural groups classified as vascular cylinder and central stem. The vascular cylinder and green and ripe fruit were used for LAB isolation. These materials were processed for analysis using standard procedures [16,17], and serial dilution aliquots (1:10, *v*/*v*; 100 μL) were plated on de Man, Rogosa, and Sharpe (MRS) agar (HiMedia, Mumbai, India) and incubated (37 ± 1 °C, 48 h) under anaerobiosis (Anaerogen, Oxoid Ltd.a., Hampshire, Wade Road, UK). At the end of the incubation period, at least five colonies with different morphologies were randomly recovered and maintained on MRS agar (4 ± 0.5 °C) [16,18].

Isolates presumptively identified as LAB (catalase-negative, non-motile, and Gram-positive cocci or rods) [17] were stored (−20 ± 1 °C) in MRS broth (HiMedia) with glycerol (Sigma Aldrich, St. Louis, MA, USA; 15 mL/100 mL) and submitted for a pre-identification step with a matrix-assisted laser desorption/ionization-time of flight mass spectrometry (MALDI-ToF MS) technique [18]. Approximately 20 colonies of each isolate grown anaerobically on MRS agar for 48 h (37 ± 1 °C) were resuspended in 1.2 mL of 75% ethanol (Química Moderna, Barueri, SP, Brazil). After centrifugation (14,000× *g*, 2 min, 4 °C) and the removal of the supernatant, cell proteins were extracted with 50 µL of acetonitrile– formic acid–water solution (50:35:15, *v*/*v*) using stirring with a Vortex (Tecnal, Piracicaba, SP, Brazil) for 1 min. The supernatant (1 μL) was deposited in two wells of a sample plate, dried at room temperature (25 ± 0.5 °C), and overlaid with 1 μL of saturated alpha-cyano-4-hydroxycinnamic acid solution (10 mg/mL) in acetonitrile–water–trifluoroacetic acid (50:47:3, *v*/*v*) (Bruker Daltonics, Germany). Measurements of MALDI-ToF MS spectra were carried out with a Bruker Biotyper 3.1 (Bruker Daltonics) and mass spectra were processed with MALDI BiotyperTM 3.1 software (Bruker Daltonics). 

Results of the pre-identification step were expressed as BioTyper log (scores) indicating the similarity of unknown MALDI-ToF MS profile with available database entries. BioTyper logs (score) of ≥2.3 and ≤3.0 indicated highly probable species identification; logs (score) of ≥2.0 and <2.3 indicated secure genus identification and probable species identification; logs (score) of ≥1.7 and <2.0 indicated probable genus identification; and logs (score) of <1.7 indicated no significant similarity between unknown profile and database entries. All isolates pre-identified as LAB were evaluated for their safety characteristics. 

### 2.2. Evaluation of Safety Characteristics

#### 2.2.1. Hemolytic Activity

The hemolytic activity was evaluated with a blood agar formulated with Mueller Hinton agar (HiMedia) and 5% (*v*/*v*) of fresh human blood. Inoculated blood agar plates (37 °C, 48 h) were examined for the appearance of β-haemolysis (clear zones surrounding colonies), α-hemolysis (green zones surrounding colonies), and γ-hemolysis (no zones surrounding colonies; no hemolytic activity) [19].

#### 2.2.2. Mucinolytic Activity

The mucin degradation was evaluated with bacteriological agar (1.2 g/100 mL) without glucose and with glucose (Sigma-Aldrich, 3 g/100 mL) with the addition of partially purified porcine gastric type III mucin (Sigma-Aldrich, 0.5 g/100 mL). Inoculated agar plates (37 ± 1 °C, 48 h) were stained with 0.1% (*w*/*v*) amido black (Sigma-Aldrich) in 3.5 mol/L of acetic acid (Merck, Darmstadt, Germany), and washed with 1.2 mol/L of acetic acid (Merck). The appearance of a clear zone surrounding the colonies was indicative of positive mucinolytic activity [20].

#### 2.2.3. Evaluation of Antibiotic Susceptibility

The LAB isolates were examined for antibiotic susceptibility with a standard disc diffusion method. The tested antibiotics were ampicillin (10 μg/disc), chloramphenicol (30 μg/disc), erythromycin (15 μg/disc), clindamycin (2 μg/disc), tetracycline (30 μg/disc), vancomycin (30 μg/disc), gentamicin (10 μg/disc), tobramicin (10 μg/disc), and streptomycin (300 μg/disc). The antibiotic susceptibility of the isolates was evaluated considering the diameter of the growth inhibition zones based on standard criteria and classified as susceptible (S) or resistant (R) [21,22]. 

The results of the experiments to evaluate the hemolytic activity, mucin degradation, and antibiotic susceptibility were considered as exclusion criteria. The isolates with mucinolytic activity, hemolytic activity, and or antibiotic resistance were excluded from further experiments. All the isolates passing the safety tests were examined for in vitro physiological functionality properties. Before use in these experiments, the isolates were activated through anaerobic cultivation (24 h, 37 ± 1 °C) in MRS broth. Bacterial suspensions with viable counts of approximately 6 log of colony-forming unities (cfu) per mL (cfu/mL) were used in these experiments [23].

### 2.3. Evaluation of In Vitro Physiological Functionality Properties

#### 2.3.1. Acid and Bile Salt Tolerance

The tolerance to low pH value and bile salt high concentration was evaluated in phosphate buffer solution (PBS, 0.05 mol/L K_2_HPO_4_/KH2PO_4_) with pH adjusted to 2 using 1 M of HCl or supplemented with 1% (*w*/*v*) bile salts (Sigma-Aldrich) after 3 h of exposure (37 ± 1 °C). The results were expressed as log cfu/mL [18]. A detection limit of 2 log cfu/mL was used in these experiments.

#### 2.3.2. Cell Surface Hydrophobicity

The cell surface hydrophobicity was evaluated in MRS broth with N-hexadecane (Sigma-Aldrich) with the absorbance reading at 560 nm. The cell surface hydrophobicity was determined with the equation:

%H = [(H_0_ − H)/H_0_] × 100 (1)

where H_0_ and H refer to the absorbance value determined before and after the extraction with N-hexadecane, respectively [24].

#### 2.3.3. Autoaggregation and Coaggregation Capacity

The cells of the isolates cultivated in MRS broth were harvested and diluted in sterile saline solution (NaCl 0.85 g/100 mL) to achieve an absorbance at 660 nm of 0.3. After a 60 min incubation at 37 ± 1 °C, the absorbance was measured again. The autoaggregation was determined with the equation:

% autoaggregation = [(A_0_ − A)/A_0_] × 100 (2)

where A_0_ refers to the initial absorbance value and A refers to the absorbance value determined after 60 min of incubation [25]. 

The method for preparing the cell suspensions for coaggregation measurement was the same used for autoaggregation measurement. The pathogenic bacteria used were *Listeria monocytogenes* (INCQS 00266, originally ATCC 7644) and *Escherichia coli* (INCQS 00219, originally ATCC 8739). The coaggregation was determined with the equation:

% coaggregation = [(OD_0_ − OD_60_)/OD_0_] × 100 (3)

where OD_0_ refers to the initial OD value determined at time zero and OD_60_ refers to the OD value of the supernatant determined after 60 min of incubation [25].

#### 2.3.4. Evaluation of the Antagonistic Activity against Pathogens

The antagonistic activity against pathogens was evaluated using an agar spot method with *E. coli* (INCQS 00219, originally ATCC 8739) and *Salmonella enterica* serovar Typhimurium (INCQS 00150, originally ATCC 14028) as indicator microorganisms. The occurrence of antagonistic activity was recorded when the diameter (mm) of the growth inhibition zones around each point (velar zone) was >1 mm [18].

### 2.4. Evaluation of In Vitro Technological Properties

#### 2.4.1. Proteolytic Activity

The proteolytic activity was evaluated with plate count agar (HiMedia) with the addition of UHT skim milk (Camponesa, Belo Horizonte, MG, Brazil; 10 g/100 mL) and an anaerobic incubation (30 ± 1 °C, 72 h). The appearance of a clear zone surrounding the colonies was indicative of positive proteolytic activity [26].

#### 2.4.2. Diacetyl Production

The diacetyl production was evaluated in UHT milk (Camponesa, 5 mL) with 0.5 mL of α-naphthol (Sigma Aldrich, 1%, *w*/*v*) and KOH (Sigma Aldrich, 16%, *w*/*v*). The formation of a red ring at the top of the inoculated milk was considered positive for diacetyl production. The diacetyl production was classified as weak (+), medium (++), and strong (+++), based on the intensity of the red ring [23].

#### 2.4.3. Exopolysaccharide Production

The isolates were cultured in MRS broth with glucose (Sigma-Aldrich, 2% *w*/*v*, 3 days, 37 ± 1 °C). The cells were collected and homogenized with 95% cold ethanol (Química Moderna) to induce exopolysaccharide (EPS) precipitation. The dry weight (mg/L) was measured to determine the amount of EPS produced [27].

#### 2.4.4. Tolerance to Sodium Chloride

MRS broth supplemented with sodium chloride (NaCl, Merck; 1, 2, 3, 4, and 5% *w*/*v*) was used to evaluate the NaCl tolerance. The results were expressed as the percent of the bacterial survival rates, i.e., the difference between the viable counts determined in MRS broth supplemented with the tested NaCl concentrations and those determined in MRS broth without NaCl (control) [23]. A detection limit of 2 log cfu/mL was used in these tests.

#### 2.4.5. Ability to Grow at Different Temperatures

The isolates were evaluated for their ability to grow at 35, 37, 40, and 45 °C in MRS broth (pH 6.8) containing bromocresol purple (Sigma Aldrich, 0.16 g/L) as a pH indicator. The inoculated broths were incubated for 48 h (35–45 °C). At the end of the incubation period, a color change from purple to yellow due to broth acidification was indicative of bacterial growth [28].

### 2.5. Selection of Isolates with the Most Promising In Vitro Characteristics for Probiotic Using Multivariate Analyze

As a first step, the results of the experiments to evaluate in vitro the physiological functionality properties (acid and bile salt tolerance, cell surface hydrophobicity, autoaggregation, coaggregation capacity, and antagonistic activity against pathogens) were used to run a principal component analysis (PCA). The most promising LAB isolates were selected to assess their technological properties (proteolytic activity, diacetyl production, EPS production, tolerance to sodium chloride, and an ability to grow at different temperatures) and an ability to survive in different food matrices. 

### 2.6. Survival of Selected Isolates in Different Food Matrices 

The ability of the most promising selected LAB isolates to survive in commercial whole and additive/preservative-free chestnut milk, whey protein drink, and mate tea with mint during 21 days of refrigeration storage was evaluated with the enumeration of the viable cell counts and measurements of the size of cell subpopulations with different physiological states using multiparametric flow cytometry. The food matrices were sterilized (121 °C, 1.1 atm, 15 min) before use in these experiments. 

A 1-mL aliquot of the tested LAB isolate cultivated (37 ± 1 °C) overnight in MRS broth under anaerobiosis was inoculated in 100 mL of each food matrix (final viable counts of approximately 7 log cfu/mL) and stored (4 ± 0.5 °C). On days 1, 7, 14, and 21 of storage, a 1-mL aliquot of the inoculated food matrix was serially diluted in sterile peptone water (1:9, 10^−1^−10^−5^) and a 100-µL aliquot was plated on MRS agar. After an anaerobic incubation (37 ± 1 °C, 48 h), the visible colonies were counted, and the results were expressed as log cfu/mL. 

Simultaneously, a staining procedure with propidium iodide (PI, Sigma-Aldrich, 10 μg/mL) and carboxyfluorescein diacetate (cFDA, Sigma-Aldrich, 2.5 μg/mL) was performed on each storage period to measure the cytoplasmic membrane integrity and enzymatic activity of the inoculated LAB cells, respectively. The staining procedures, data acquisition, and analysis were performed with a flow cytometer (BD Accuri C6, Becton Dickinson, Franklin Lakes, NJ, USA) [29,30]. The bacterial cell subpopulations characterized as PI-cFDA+ were considered as non-permeabilized cells with enzymatic activity (living cells); PI+cFDA- were considered as permeabilized cells without enzymatic activity (dead cells); and PI+cFDA+ were considered as permeabilized cells with enzymatic activity (injured cells). 

### 2.7. Identification of Selected Isolates by Sequence Analysis of 16S-rRNA Gene

The bacterial genomic DNA was extracted with a magnetic bead method (Neoprospecta Microbiome Technologies, Florianópolis, SC, Brazil). The primers 341F (CCTACGGGRSGCAGCAG) [31] and 806R (GGACTACHVGGGTWTCTAAT) were used for the detection of 16S-rRNA V3/V4 region [32]. The PCR was run with a MiSeq Sequencing System (Illumina Inc., San Diego, CA, USA), and reactions were taken using a V2 kit, 300 cycles, and single-end sequencing. The sequences were analyzed with a proprietary pipeline (Neoprospecta Microbiome Technologies). The reads were individually submitted to a quality filter considering the sum of the DNA base probability errors, which allowed a maximum of 1% accumulated errors. The DNA sequences corresponding to Illumina adapters were removed. The resulting sequences with 100% identity were clustered for use in taxonomic identification with accurate 16S-rRNA sequences database NeoRef (Neoprospecta Microbiome Technologies). The bacterial identification was assumed when the query sequence had >99% similarity to the 16S-rRNA gene sequence. 

### 2.8. Statistical Analysis

The experiments were carried out in three biological replicates and three technical replicates. ANOVA followed by Tukey’s test was used to determine significant differences among the results (average ± standard deviation). A *p*-value of <0.05 was considered statistically significant. The results of the survival of the selected isolates in different food matrices were analyzed in a second PCA. The RStudio software was used to run the statistical analysis [33]. 

## 3. Results

### 3.1. Isolation and Preliminary Identification of Isolates 

A total of 122 presumptive mesophilic and microaerophilic isolates were recovered from xique-xique cladodes (vascular cylinder) and fruit (green and ripe). These isolates were randomly picked from MRS agar according to the apparent differences in morphological characteristics of the colonies (e.g., size, edge, color, and topography). Sixty isolates were recovered from vascular cylinder samples, 34 were recovered from ripe fruit samples, and 28 were recovered from unripe fruit. Seventeen isolates had typical characteristics of LAB, to cite: Gram-positive, catalase-negative, non-motile, non-spore-forming rod-cocci and cocci. The MALDI-ToF MS technique reached a species-level identification of these 17 isolates, which were pre-identified as *Lacticaseibacillus paracasei* (six from unripe fruit, seven from ripe fruit, and four from vascular cylinder). The MALDI-ToF MS scores of identifications were in the range of 2.07–2.23.

### 3.2. Safety Analysis

The 17 preliminarily identified LAB isolates were evaluated with safety tests. All the isolates positive for β-hemolysis and or mucinolytic activity were excluded and remaining eight isolates were analyzed in the antibiotic susceptibility test. Only two isolates were resistant to clindamycin and ampicillin (isolates 79 and 80) (Table 1). Six isolates sensitive to all the examined antibiotics were included in the further experiments. 

### 3.3. Selection of LAB Isolates Based on In Vitro Physiological Functionality Characteristics 

All LAB isolates presented tolerance to the exposure to 1% bile salt concentration for 3 h (viable counts: 7.3 ± 0.2–4.9 ± 0.2 log cfu/mL). However, the isolates showed sharp decreases in viable counts when they were exposed to pH 2 for 3 h. Only isolate 98 showed viable counts of >2 log cfu/mL. The isolates showed cell surface hydrophobicity values varying from 4.3 to 22.9%; the highest and lowest surface hydrophobicity values were found for isolates 98 and 84, respectively. All isolates had the ability to auto aggregate and coaggregate. The highest values for auto-aggregation were found for isolate 69 (28.0 ± 0.2%), while the highest values for coaggregation with *L. monocytogenes* and *E. coli* were found for isolate 98 (19.4 ± 0.6 and 17.8 ± 0.5%, respectively). All isolates had antagonistic activity against *E. coli* and *S.* Typhimurium, with diameters of growth inhibition zones in the range of 3–8 mm (Table S1).

**Figure 1:**
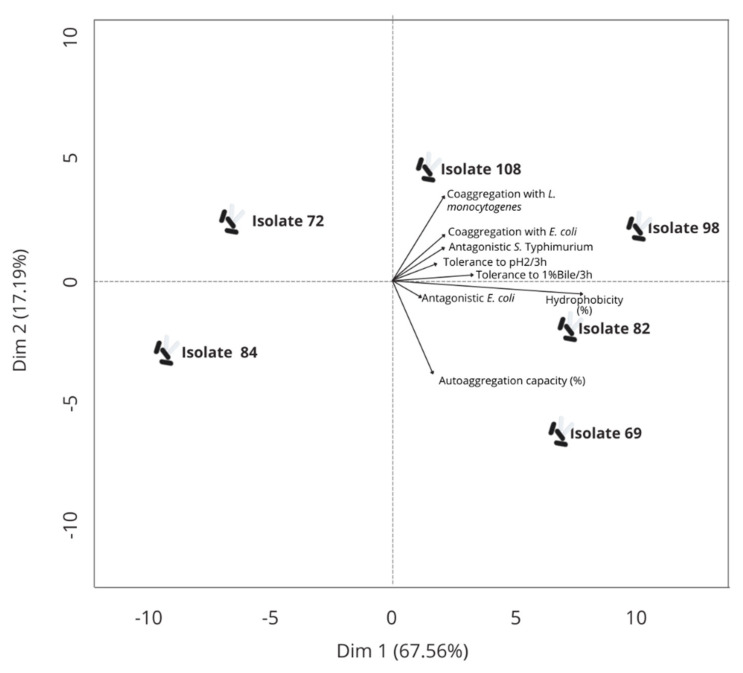
Principal component analysis (PCA) run for lactic acid bacteria isolates from xique-xique cladodes and fruit showing the variable projection to acid tolerance, survival with bile salts, hydrophobicity, auto-aggregation, coaggregation capacity, and antagonist activity.

The first PCA was run to select the isolates with the best performances in tests to evaluate in vitro the probiotic-related physiological functionalities (Figure 1). The isolates 69, 82, 98, and 108 located at the right upper and lower quadrant had the best results overall in these tests (acid tolerance, survival with bile salts, hydrophobicity, auto-aggregation capacity, coaggregation, and antagonist activity), being selected for use in further investigations.

### 3.4. Technological Properties and Survival of Selected Isolates in Different Food Matrices 

All the examined isolates (69, 82, 98, and 108) were positive for proteolytic activity and capable of producing diacetyl and of growing at 35, 37, 40, and 45 °C. All the isolates were also capable of producing EPS (75–90 mg/L). The highest and lowest EPS production rates were found for isolates 82 and 69, respectively. The examined isolates showed good tolerance to 1, 2, and 3% NaCl, with survival rates in the range of 86.2 to 99.5%. Only isolate 69 decreased the survival rate to 20.1% following the exposure to 4% NaCl. The survival rates of the examined isolates were in the range of 11.5 to 52.8% following the exposure to 5% NaCl. The highest survival rates following the exposure to 5% NaCl were found for isolate 98 (Table 2).

All the isolates had increased (*p* < 0.05) or maintained similar (*p* ≥ 0.05) viable counts in the distinct examined food matrices during the 21 days of refrigeration storage. The isolates had viable counts of >9 log cfu/mL in the chestnut milk and the whey protein drink on day 21 of storage. The lowest viable counts of the isolates on day 21 of storage were found in mate tea with mint (7.3 ± 0.8–8.5 ± 0.5 log cfu/mL). The highest and lowest viable counts (*p* < 0.05) on day 21 of storage were found for isolate 108 in chestnut milk (9.5 ± 0.3 log cfu/mL) and mate tea with mint (7.3 ± 0.8 log cfu/mL), respectively (Table S2).

The results of the multiparametric flow cytometry analysis showed that all isolates had high sizes of PI-cFDA+ cell subpopulations (39.4 ± 1.4–61.9 ± 0.5%) in the chestnut milk and the whey protein drink on day 21 of refrigeration storage, which are characterized as metabolically active living cells. The isolates 82 and 108 had the highest sizes of PI-cFDA+ cell subpopulations (61.9 ± 0.5% and 51.6 ± 0.6%, respectively) in the whey protein drink on day 21 of storage. All examined isolates had low sizes of PI-cFDA+ cell subpopulations in the mate tea with mint (0.3 ± 0.5–11.1 ± 0.4%) on day 21 of storage (Table S2). 

Low sizes of PI+cFDA- cell subpopulations (characterized as dead cells) were found for all isolates in the chestnut milk and the whey protein drink (0.2 ± 0.1–4.4 ± 0.5%) on day 21 of storage. The higher sizes of PI+cFDA- cell subpopulations were found in the mate tea with mint (17.9 ± 0.9–95.6 ± 0.3%). The isolates had decreases (*p* < 0.05) in sizes of PI+cFDA+ cell subpopulations (characterized as injured but still functioning cells) during storage in the whey protein drink (varying from 4.7 ± 0.5 to 30.2 ± 0.7%) and the chestnut milk (varying from 3.0 ± 0.1 to 24.4 ± 0.5%). However, isolates 69 and 98 had increases (*p* < 0.05) (varying from 25.0 ± 0.6 to 75.4 ± 0.4%) and isolates 82 and 108 had decreases (*p* < 0.05) (varying from 0.9 ± 0.4 to 57.3 ± 0.3) in sizes of PI+cFDA+ cell subpopulations in the mate tea with mint during storage (Table S2). 

**Figure 2:**
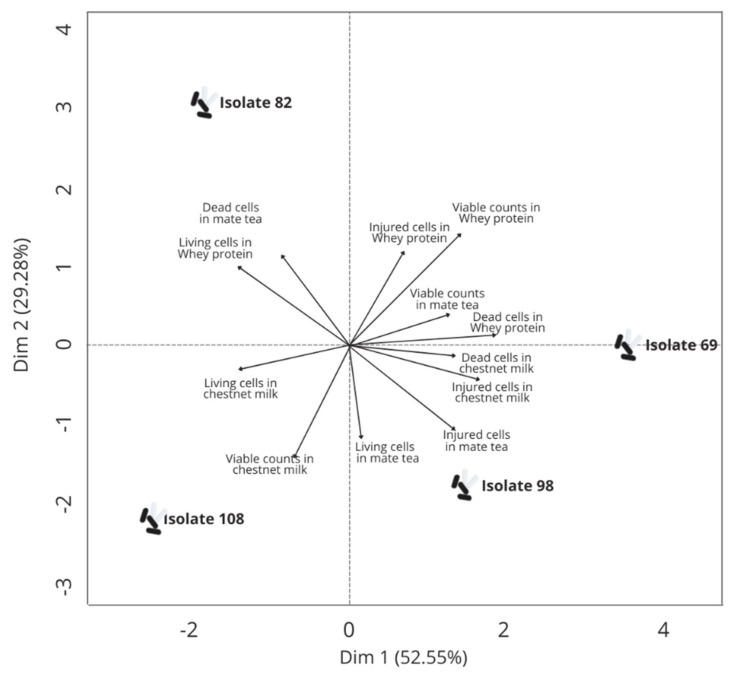
Principal component analysis (PCA) run of the variable projection to the survival of lactic acid bacteria isolates from xique-xique cladodes and fruit selected as having the most promising probiotic-related characteristics in different food matrices on day 21 of refrigeration storage.

A second PCA (Figure 2) was run to assess the results of the experiments to measure the survival of the selected isolates (69, 82, 98, and 108) in the different examined food matrices. The isolate 82 located at the left upper quadrant had the better survival results in the whey protein drink. The isolate 108 located at the left lower quadrant had better survival results in the chestnut milk and isolates 69 and 98 located at the right had better survival results in the matte tea with mint. 

The isolates 69, 82, and 98 were identified by 16S-rRNA sequencing as being *Lacticaseibacillus paracasei* (coded as *L. paracasei* 69, *L. paracasei* 82, and *L. paracasei* 98, respectively), while the isolate 108 was identified as *Lacticaseibacillus casei* (coded as *L. casei* 108). The sequences were deposited into the Genbank nucleotide sequence database. The genera of these four isolates identified by 16S-rRNA sequencing agreed with MADI-ToF MS pre-identification, while the species of only two isolates agreed.

## 4. Discussion

This is the first study using the MALDI-TOF MS technique to perform a preliminary identification of autochthonous LAB isolated from the cactus xique-xique. MALDI-TOF MS is considered a powerful, fast, reliable, and cost-effective technique for the identification of LAB [34]. This technique was used to select only isolates pre-identified as belonging to LAB genus, and all the isolates were pre-identified as *L. paracasei* with high scores. 

Each type of vegetable and fruit provides a dominant autochthonous microbiota where LAB represent only a small portion of these microorganisms [5]. The species most frequently isolated from vegetables and fruit belong to the genera *Lactobacillus*, *Leuconostoc*, *Enterococcus*, *Weissella*, and *Pediococcus* [5]. Previous studies found *Leuconostoc mesenteroides*, *Lactiplantibacillus plantarum*, *Weissella cibaria*, and *Fructobacillus fructosus* as part of the autochthonous microbiota of cacti [7,35,36]. 

This study performed a screening of LAB isolates from xique-xique cladodes and fruit using a sequence of in vitro security and physiological functionality analysis to select the isolates with the most promising probiotic-related aptitudes, which were evaluated for their technological properties and capacity of surviving in different food matrices. First, the isolates were evaluated regarding their safety characteristics, including hemolytic and mucinolytic activities since the absence of these activities is a prerequisite to characterize a bacterial isolate as probiotic [23,37]. *Lactobacillus* (and recently amended genera [38]) species are typically non-hemolytic and rarely some studies have found partial hemolytic activity in *Lactobacillus* isolates/strains. The production of mucin-degrading enzymes is considered a virulence factor for enteropathogens and an undesirable characteristic for probiotics since it can favor the alteration of the intestinal mucosal barrier [23]. Therefore, the isolates showing mucinolytic activity were excluded. 

According to the “qualified presumption of safety” (QPS) concept, the presence of antibiotic resistance in LAB is an important safety criterion [39] since LAB could offer antimicrobial resistance genes to enteric or foodborne pathogens [40]. Thus, two isolates presenting resistance to clindamycin and ampicillin were excluded from this study and six isolates were evaluated concerning their in vitro probiotic-related physiological properties. 

The high acidity (pH < 3) in the gastric environment and high concentration of bile salt components in the proximal intestine are the most important characteristics affecting the survival and functionality of probiotics after ingestion. The ability to tolerate these conditions allows the probiotics to survive and colonize the gastrointestinal tract [7]. Moreover, most of the probiotics could exhibit unpredictable resistance to acidic conditions, which is a species and isolate/strain-dependent feature [1]. All the examined LAB isolates had good abilities to survive to the high bile salt concentration, although they showed lower viable counts following a 3-h exposure to a very acidic pH (pH 2). 

All the examined LAB isolates showed high rates of cell surface hydrophobicity, auto-aggregation, and coaggregation capacities. Some cell surface properties, specifically the hydrophobicity and auto-aggregation, were evaluated to obtain putative responses related to the capacity of the LAB isolates of adhering to the intestinal mucosa. Moreover, the coaggregation ability helps to form a barrier to prevent pathogen colonization [1]. All isolates exerted inhibitory effects against *E. coli* and *S.* Typhimurium. The antagonistic activity against pathogens is an important functional property of probiotics, as they are linked to their capability of producing antimicrobial compounds [23].

Only one previous study evaluated the probiotic properties of autochthonous LAB isolated from cactus (*Opuntia ficus-indica*) fruit and the isolates selected as having the most promising probiotic-related aptitudes were identified as *L. plantarum* and *F. fructosus* [7]. The use of multivariate approaches has been tested for the screening of LAB isolates from various raw materials for distinct applications. The PCA combines a qualitative measure of the diversity (represented as a dispersion of the LAB isolates in the factorial space) with the projection of input variables representing an overview of the most important factors that account for the data dispersion [41]. The PCA selected four LAB isolates (isolates 69 and 98 recovered from green fruit, isolate 82 recovered from ripe fruit, and isolate 108 recovered from vascular cylinder) as having the most promising probiotic-related in vitro physiological characteristics (tolerance to acidic pH and bile salts, hydrophobicity, auto-aggregation, coaggregation, and antagonistic activity).

These selected LAB isolates were examined considering a set of technological properties and survival in different food matrices. All the isolates had proteolytic activity and the capacity of producing diacetyl. Probiotic LAB with proteolytic activity have been associated with higher levels of soluble proteins and delivery of free amino acids into food matrices [42]. Diacetyl exerts an important role in the development of flavor in various food products, with the production of distinguished sensory characteristics [23]. An important characteristic for LAB isolates intended for incorporation into food matrices is their ability to tolerate different NaCl concentrations since the NaCl concentrations commonly used in food are inhibitory to these microorganisms [43]. Overall, all examined isolates tolerated concentrations of up to 5% of NaCl, besides having a strong ability to produce EPS. Increasing attention has been given to the EPS production by probiotics because this component could be related to the rheological properties of food products, besides causing improved immunogenic properties and beneficial modulation of gut microbiota in the host [23].

In addition to having good technological characteristics, the four selected isolates had ability to survive with high viable counts and large sizes of physiologically active cell subpopulations in distinct food matrices still few exploited for the delivery of probiotics. LAB isolated from plant matrices have commonly shown the ability to survive under simulated conditions of the digestive tract, as well as in food matrices with conditions considered typically not ideal for keeping the viability of probiotics [18,23]. 

Some characteristics related to a delivery matrix considered ideal to produce probiotic food could be having a pH closer to neutrality, a good buffering capacity, a source of nutrients, and an ability to avoid oxygen access to the food surface [44]. The best survival of the selected isolates during refrigeration storage was found in the chestnut milk and the whey protein drink, while the lower survival was found in the mate tea with mint. On the contrary to our results, an early study reported that yerba mate, the raw material to produce mate tea, exerted stimulatory effects on the growth of probiotic LAB in vitro [45]. Despite these results, the three selected isolates had high viable counts (≥7 cfu/mL) in the chestnut milk, the whey protein milk, or the mate tea with mint at the end of the 21 days of refrigeration storage. Distinct specific viable count levels are suggested as the minimum number of viable cells per gram for a given portion of probiotic foods. However, for specific health claims, probiotics in food must prove the delivery of an effective dose at the end of the shelf life, which could vary among strains [15]. 

The conditions found in some food products should cause physiological damage in probiotic cells; thus, it is important to select probiotic isolates/strains capable of tolerating these conditions or even of adapting and recovering from induced damage. Flow cytometry technique has been used to investigate alterations in the viability and physiological status of probiotic cells in foods, giving valuable information on the heterogeneity of viable and non-viable subpopulations in delivery matrices [46,47]. The high viable counts and low-to-moderate percentage (<45%) of non-viable (dead) cells of selected isolates in the chestnut milk, the whey protein drink, and the mate tea with mint at the end of the 28 days of refrigeration storage indicate their capacity to keep vitality (physiological/metabolic functionalities) when incorporated into different foods, representing positive characteristics for probiotic LAB [48].

The four isolates selected as having the most promising probiotic-related in vitro aptitudes were identified by 16S-rRNA sequencing as *L. paracasei* (*L. paracasei* 69, *L. paracasei* 82 and *L. paracasei* 98), or *L. casei* (*L. casei* 108), which are LAB species with a long and safe use as probiotics in a variety of food or supplements [49,50]. These results reinforce the possibility of exploiting these isolates in different strategies for the development of probiotic foods with distinct technological characteristics. *L. casei* 108 were distinctly identified in MALDI-TOF MS and 16S-rRNA gene analysis. An early study also found divergences between MALDI-TOF MS and 16S rRNA gene sequence analysis methods for the identification of *L. casei* and *L. paracasei* [18]. This difference could be linked to the number of species (*L. casei*, *L. paracasei*, *L. rhamnosus* and *L. zeae*) forming the *L. casei* group, which cannot be distinguished by conventional phenotypic properties [51,52]. In general, MALDI-TOF MS and 16S rRNA techniques have shown high congruency in the identification of LAB species [18].

## 5. Conclusions

The results showed that xique–xique cladodes and fruit are sources of LAB isolates with in vitro characteristics compatible with probiotic use, in addition to showing in vitro technological properties that could be exploited in the formulation of probiotic foods with particular characteristics. The four LAB isolates selected as having the most promising probiotic aptitudes were identified as *L. paracasei* or *L. casei* and had the capability of surviving and keeping physiological functions in food formulations with distinct characteristics. Further in vivo studies should investigate the specific health-promoting effects of these isolates and confirm their definitive potential for use as novel probiotics.

## Figures and Tables

**Table 1:** Results of antibiotic susceptibility profile of lactic acid bacteria isolates from xique-xique cladodes and fruit.

Isolate	Chloramphenicol (30 μg/Disc)	Erythromycin (15 μg/Disc)	Tobramycin (10 μg/Disc)	Tetracycline (30 μg/Disc)	Clindamycin (2 μg/Disc)	Ampicillin (10 μg/Disc)	Vancomycin (30 μg/Disc)	Gentamycin (10 μg/Disc)	Streptomycin (300 μg/Disc)
69	S	S	S	S	S	S	S	S	S
72	S	S	S	S	S	S	S	S	S
79	S	S	S	S	R	S	S	S	S
80	S	S	S	S	S	R	S	S	S
82	S	S	S	S	S	S	S	S	S
84	S	S	S	S	S	S	S	S	S
98	S	S	S	S	S	S	S	S	S
108	S	S	S	S	S	S	S	S	S

S: Susceptible; R: Resistant.

**Table 2:** Results of in vitro tests to measure the technological properties of lactic acid bacteria isolates from xique-xique cladodes and fruit selected as having the most promising probiotic-related characteristics.

Isolate	Diacetyl Production *	Proteolytic **	**EPS Production (mg/L)**	Capability to Grow at Different Temperatures (°C) **					**Tolerance to NaCl**				
				**35**	**35**	**37**	**40**	**45**	**1%**	**2%**	**3%**	**4%**	**5%**
69	++	+	75.1 ± 1.5 ^d^	+	+	+	+	+	89.5 ± 0.9 ^a^	86.2 ± 0.6 ^b^	88.6 ± 0.3 ^c^	20.1 ± 0.1 ^c^	17.3 ± 0.3 ^c^
82	++	+	90.6 ± 0.9 ^a^	+	+	+	+	+	99.5 ± 0.3 ^a^	99.3 ± 0.2 ^a^	96.8 ± 0.4 ^a^	94.4 ± 0.6 ^a^	11.5 ± 0.4 ^d^
98	++	+	88.1 ± 0.4 ^b^	+	+	+	+	+	98.9 ± 0.1 ^a^	97.8 ± 0.9 ^a^	90.4 ± 0.1 ^b^	87.7 ± 0.1 ^b^	52.8 ± 0.1 ^a^
108	++	+	78.3 ± 0.3 ^c^	+	+	+	+	+	99.3 ± 0.7 ^a^	99.2 ± 0.3 ^a^	95.7 ± 0.7 ^a^	85.9 ± 0.7 ^b^	28.4 ± 0.1 ^b^

EPS: Exopolysaccharide production. * Diacetyl production: (+) weak, (++) medium; ** (+) Positive proteolytic activity or capability of growing at the tested temperature. Different superscript small letters in the same column denote differences (*p* < 0.05) among the different tested isolates, based on Tukey’s test.

## Data Availability

All data generated or analyzed during this study are included in this published article.

## Supplementary Materials

The following are available online at https://www.mdpi.com/article/10.3390/foods10122960/s1, Table S1: Physiological functionality properties of lactic acid bacteria isolated from xique-xique cladodes and fruit and Table S2: Viable counts (cfu/mL) and size (%) of subpopulations of the four isolates selected as having the most promising probiotic-related characteristics in different foods during 21 days of refrigeration storage (4 ± 0.5 °C).
