# Supplementary material for: Selection of Lactic Acid Bacteria with In Vitro Probiotic-Related Characteristics from the Cactus Pilosocereus gounellei (A. Weber ex. K. Schum.) Bly. ex Rowl
Source: Foods. 2021 Dec 1;10(12):2960. doi: 10.3390/foods10122960 (PMC8700760; doi:10.3390/foods10122960)
Supplement: Supplementary file 1 [file foods-10-02960-s001.zip › foods-1448747-supplementary.pdf]

Table S1. Results of *in vitro* physiological-related functionality properties of distinct lactic acid bacteria isolates from xique-xique cladodes and fruit.

Isolate	Bile salt concentration 1% (3h) (cfu/mL)*	pH 2 (3h) (cfu/mL)*	Hydrophobicity (%)	Autoaggregation (%)	Coaggregation (%)		Antagonistic activity (Diameter of growth inhibition zones; mm)	
					L. monocytogenes	*E. coli*	*E. coli*	*S.* Typhimurium
69	5.2 ± 0.5[c]	<2 ± 0.0[b]	20.3 ± 1.4[b]	28.0 ± 0.2[a]	9.1 ± 0.1[c]	14.8 ± 1.5[b]	8.0 ± 0.3[a]	4.0 ± 0.1[b]
72	4.9 ± 0.2[c]	<2 ± 0.0[b]	8.5 ± 1.3[d]	21.3 ± 0.9[c]	12.9 ± 2.1[b]	16.2 ± 2.1[ab]	6.0 ± 0.5[b]	4.0 ± 0.2[b]
82	7.1 ± 0.1[a]	<2 ± 0.0[b]	18.9 ± 0.4[b]	23.1 ± 1.3[b]	12.7 ± 2.9[b]	15.2 ± 0.2[b]	8.0 ± 0.7[a]	3.0 ± 0.5[c]
84	6.8 ± 0.1[b]	<2 ± 0.0[b]	4.3 ± 1.3[e]	27.2 ± 4.3[ab]	12.8 ± 1.3[b]	12.7 ± 0.3[c]	6.0 ± 0.4[b]	3.0 ± 0.2[c]
98	7.3 ± 0.2[a]	2.5 ± 0.3[a]	22.9 ± 1.0[a]	27.1 ± 3.1[ab]	19.4 ± 0.6[a]	17.8 ± 0.5[a]	6.0 ± 0.2[b]	4.0 ± 0.3[b]
108	6.3 ± 0.3[b]	<2 ± 0.0[b]	14.9 ± 0.2[c]	19.5 ± 2.0[c]	14.4 ± 1.7[b]	15.6 ± 0.7[b]	7.0 ± 0.8[ab]	5.0 ± 0.4[a]

*Survival (viable counts) when exposed to this condition.

Different superscript small letters in the same column denote differences (P <0.05) among the different tested isolates, based on Tukey's test.

Table S2. Viable cell counts (cfu/mL) and sizes (%) of cell subpopulations of the 4 lactic acid bacteria isolates (69, 82, 98, and 108) selected as having the most promising *in vitro* probiotic-related characteristics in different foods during 21 days of refrigeration storage (4 ± 0.5 °C).

		Chestnut milk				Mate tea with mint				Whey protein drink			
Isolate	Days of storage	PI-cFDA+	PI+cFDA+	PI+cFDA-	Viable counts (cfu/mL)	PI-cFDA+	PI+cFDA+	PI+cFDA-	Viable counts (cfu/mL)	PI-cFDA+	PI+cFDA+	PI+cFDA-	Viable counts (cfu/mL)
	1	27.9±0.6^{c}	14.8±0.2^{b}	3.2±0.2^{b}	7.9±0.1^{b}	1.6±0.3^{c}	62.5±0.6^{b}	32.2±0.8^{b}	7.4±0.6^{a}	20.5±0.1^{c}	9.8±0.6^{a}	11.2±0.1^{a}	7.9±0.3^{b}
	7	35.7±0.4^{b}	16.7±0.3^{a}	4.5±0.3^{a}	8.7±0.5^{a}	0.5±0.1^{d}	51.0±0.8^{c}	47.3±0.1^{a}	7.8±0.8^{a}	37.9±0.6^{a}	6.5±0.1^{c}	2.7±0.9^{b}	8.3±0.1^{b}
	14	41.2±0.3^{a}	10.9±0.9^{d}	2.3±0.1^{c}	9.2±0.5^{a}	8.3±1.1^{a}	46.5±0.3^{d}	31.2±1.3^{b}	8.1±0.4^{a}	37.6±0.9^{a}	4.7±0.5^{d}	3.6±0.6^{b}	9.4±0.2^{a}
69	21	39.4±1.4^{a}	13.7±0.6^{c}	4.4±0.5^{a}	9.1±0.3^{a}	2.6±0.2^{b}	75.4±0.4^{a}	18.1±0.8^{c}	8.2±0.5^{a}	41.1±0.2^{b}	7.6±0.2^{b}	3.3±0.8^{b}	9.3±0.3^{a}
	1	38.4±0.1^{d}	6.9±0.1^{b}	0.6±0.1^{b}	8.6±0.9^{a}	19.8±0.8^{a}	15.3±0.7^{c}	16.7±0.6^{d}	7.7±0.3^{a}	2.7±0.8^{d}	30.2±0.7^{a}	50.1±1.2^{a}	7.9±0.9^{a}
	7	66.0±0.9^{a}	20.9±0.8^{a}	0.6±0.2^{b}	8.6±0.6^{a}	10.1±0.9^{b}	27.2±0.8^{b}	36.0±0.8^{b}	8.3±0.5^{a}	41.1±1.1^{b}	12.9±0.9^{b}	4.5±0.3^{c}	8.1±0.9^{a}
	14	40.2±0.6^{c}	6.1±0.3^{b}	0.8±0.1^{a}	8.8±0.5^{a}	9.7±0.3^{c}	54.4±1.2^{a}	26.7±0.8^{c}	7.8±0.2^{a}	29.4±0.6^{c}	6.0±0.5^{d}	6.0±0.1^{b}	8.8±0.8^{a}
82	21	46.2±0.3^{b}	3.0±0.1^{c}	0.2±0.1^{c}	9.1±0.7^{a}	0.3±0.5^{d}	0.9±0.4^{d}	95.6±0.3^{a}	7.8±0.1^{a}	61.9±0.5^{a}	8.2±0.1^{c}	1.4±0.1^{d}	9.3±0.6^{a}
	1	18.1±0.1^{c}	4.9±0.8^{c}	1.2±0.3^{b}	7.7±0.2^{b}	23.5±0.6^{b}	25.0±0.6^{d}	14.2±0.1^{c}	7.3±0.1^{b}	14.2±0.1^{d}	15.9±0.5^{a}	20.1±0.9^{a}	7.8±0.2^{c}
	7	45.6±0.6^{b}	24.4±0.5^{a}	3.2±0.6^{a}	8.7±0.3^{a}	1.2±0.3^{d}	53.9±1.1^{b}	42.6±0.8^{a}	8.3±0.3^{a}	36.6±0.5^{b}	7.3±0.9^{c}	3.1±0.3^{c}	8.3±0.1^{b}
	14	80.4±0.3^{a}	7.4±0.3^{b}	0.7±0.1^{c}	8.8±0.6^{a}	33.2±0.1^{a}	40.3±0.5^{c}	10.4±0.1^{d}	8.3±0.3^{a}	34.1±0.1^{c}	13.6±0.1^{b}	6.3±0.6^{b}	9.5±0.2^{a}
98	21	46.1±0.7^{b}	3.9±0.9^{c}	0.4±0.1^{d}	9.1±0.8^{a}	11.1±0.4^{c}	58.5±0.5^{a}	17.9±0.9^{b}	8.5±0.5^{a}	44.4±0.3^{a}	8.0±0.9^{c}	1.4±0.1^{d}	9.2±0.3^{a}
	1	36.3±0.3^{d}	8.2±0.3^{c}	1.7±0.5^{a}	7.9±0.1^{c}	1.8±0.3^{d}	57.3±0.3^{a}	33.3±0.8^{c}	7.0±0.9^{a}	17.7±0.9^{c}	13.1±0.9^{a}	13.5±0.7^{a}	7.0±0.1^{c}
	7	55.6±0.5^{b}	18.4±0.8^{a}	2.3±0.7^{a}	8.5±0.2^{b}	2.9±0.1^{c}	45.7±0.8^{b}	43.6±0.6^{a}	7.8±0.6^{a}	51.0±0.6^{a}	12.0±0.7^{a}	3.9±0.5^{c}	8.3±0.3^{b}
	14	58.8±0.7^{a}	14.8±0.1^{b}	1.6±0.8^{a}	8.6±0.1^{b}	6.3±0.2^{a}	57.1±0.4^{a}	29.5±0.3^{d}	7.9±0.3^{a}	31.6±0.2^{b}	10.8±0.1^{b}	6.9±0.5^{b}	9.4±0.3^{a}
108	21	49.0±0.6^{c}	4.0±0.3^{d}	0.4±0.3^{b}	9.5±0.3^{a}	4.3±0.1^{b}	39.6±0.1^{c}	39.5±0.1^{b}	7.3±0.8^{a}	51.6±0.6^{a}	5.4±0.1^{c}	1.3±0.1^{d}	9.2±0.1^{a}

PI-cFDA+: non-permeabilized cells with enzymatic activity (living cells); PI+cFDA-: permeabilized cells without enzymatic activity (dead cells); PI+cFDA+: permeabilized cells with enzymatic activity (injured cells).

Different superscript small letters in the same column indicate significant differences for the same isolate and food matrix at different storage periods, based on Tukey's test ($P < 0.05$).
